# Acute Pancreatitis With Normal Amylase and Lipase: A Diagnostic Dilemma

**DOI:** 10.7759/cureus.62374

**Published:** 2024-06-14

**Authors:** Hadiza Ibrahim, Adil Jumani, Khalid Elhassan, Mira Ibrahim, Majdi AlNajjar

**Affiliations:** 1 Internal Medicine, Zayed Military Hospital, Abu Dhabi, ARE; 2 Endocrinology, Zayed Military Hospital, Abu Dhabi, ARE

**Keywords:** euglycemic diabetic ketoacidosis, empagliflozin, dapagliflozin, dpp4 pancreatitis, dpp4-i, sglt-2 inhibitor, precipitating factors for euglycemic dka, hypertriglyceridemia (htg), acute pancreatitis (ap), hypertriglyceridemia-induced acute pancreatitis

## Abstract

Acute pancreatitis is a common and potentially life-threatening condition. It is characterized by inflammation of the pancreas, most often leading to elevated levels of pancreatic enzymes in the blood. In a subset of patients, however, conventional biomarker levels may remain within the reference range. Such instances have the potential to create a diagnostic challenge for healthcare professionals and can lead to misdiagnosis or delayed treatment. This article presents the intriguing clinical scenario of acute pancreatitis with normal amylase and lipase, discusses factors that may lead to normoenzymatic presentation, and reminds clinicians of the diagnostic criteria for acute pancreatitis, which does not necessarily require elevated pancreatic enzymes.

## Introduction

Acute pancreatitis is a common [1], potentially life-threatening condition with various etiologies, characterized by inflammation and destruction of the pancreas, and is commonly accompanied by elevated levels of pancreatic enzymes [2,3], most commonly tested being amylase and lipase, in the blood. These enzymes are typically used as diagnostic markers for acute pancreatitis; however, the diagnosis of acute pancreatitis requires only two out of three criteria to be met, including (1) abdominal pain consistent with the disease (typically acute onset, persistent, severe, epigastric pain usually radiating to the back), (2) serum amylase or lipase >3 times the upper limit of normal, and (3) characteristic findings on abdominal imaging (usually a contrast-enhanced CT scan or MRI) [4]. We report a case of hypertriglyceridemia-induced acute pancreatitis, which is intriguing because serum amylase and lipase were within the reference range.

## Case presentation

A 39-year-old male presented to the emergency department for the third time in two days with the same complaint: abdominal pain that had now been persistent for the past three days. He had a relatively sudden onset of moderate to severe epigastric pain; he described it as sharp, radiating to his back, worse when he lay flat, better when sitting forward or standing, and it was not much related to food. The pain was persistent and even getting worse despite two prior emergency room visits and using over-the-counter paracetamol. He had associated nausea and poor appetite since the onset of pain but denied any vomiting, diarrhea, or constipation. He had some subjective fever, which was never measured or recorded in the hospital during any of his three visits. He otherwise had no complaints and no genitourinary or respiratory symptoms. This was his first time experiencing this type of abdominal pain.

His past medical history was significant for type 2 diabetes, for which he was on sitagliptin, metformin, and dapagliflozin for a month prior to presentation. He had no prior surgeries or hospitalizations, and he had no history of gallstones or alcohol use, but he was an active smoker. His family history was non-contributory. His physical examination was significant for voluntary guarding and tenderness on palpation over his epigastrium, but his abdomen was soft and all vital signs were within normal limits.

Laboratory investigations (Table 1) revealed a raised WBC count (16 × 109/L) with neutrophil predominance and a high CRP (195). Serum amylase and lipase were within normal limits, as were his renal function tests and liver enzymes. His random blood glucose was 8.3 mmol/L, he had normal serum calcium, and no lipid profile was ordered initially. His urine was positive for ketones (4+), and his venous blood gas was acidotic with a pH of 7.29 and a high anion gap of 16. Ultrasound abdomen showed some fatty infiltration of the liver but normal gall bladder and biliary tract without stones or sludge. His diabetes was poorly controlled, as evidenced by a glycosylated hemoglobin (HbA1c) level of 11.9%.

**Table 1:** Blood tests during admission with their reference ranges ** Serum reported grossly "lipemic," marked interference with lab results cannot be ruled out; CRP: C-reactive protein, H: high above normal range, L: low above normal range, WBC: white blood cell count

Test	Normal values	Admission/day 0 **	Day 1	Day 2
Total WBC	4.5-11.0 x 10*9 cells/L	16.12 x 10*9 (H)	12.71 x 10*9 (H)	10 x 10*9
Neutrophils	1.8-5.4 x 10*9 cells/L	11.8 x 10*9 (H)	8.4 x 10*9 (H)	4.4 x 10*9
Lymphocytes	1.3-3.0 x 10*9 cells/L	3.24 x 10*9 (H)	3.05 x 10*9 (H)	4.63 x 10*9 (H)
Monocytes	0.3-0.8 x 10*9 cells/L	0.76 x 10*9	0.92 x 10*9	0.60 x 10*9
Eosinophils	0.0-0.4 × 10*9 cells/L	0.19 x 10*9	0.23 x 10*9	0.32 x 10*9
Hemoglobin	140-180 × 10*9 cells/L	165 x 10*9	157 x 10*9	160 x 10*9
Platelets	140-440 x 10*9 cells/L	248 x 10*9	243 x 10*9	246 x 10*9
Sodium	135-147 mmol/L	126 (L)	134 (L)	137
Potassium	3.5-5.0 mmol/L	4.9	4.8	4.3
Bicarbonate	22-30 mmol/L	12 (L)	19 (L)	21 (L)
Chloride	98-107 mmol/L	98	101	104
Glucose	3.6-6.0 mmol/L	8.31 (H)	8.30 (H)	6.64 (H)
Urea	2.8-7.1 mmol/L	3.6	3.3	3.7
Creatinine	44-106 umol/L	64.55	71.62	60.13
Calcium	2.1-2.6 mmol/L	2.3	2.3	2.16
Total protein	60-80 g/L	101 (H)	86 (H)	71 (H)
Albumin	35-45 g/L	36	35	42
Aspartate aminotransferase	5-34 U/L	see comment**	see comment**	22
Alanine aminotransferase	0-55 U/L	see comment**	see comment**	52
Alkaline phosphatase	30-120 U/L	123	118	104
Amylase	25-125 U/L	64	-	-
Lipase	0-60 U/L	42	-	-
Total Bilirubin	2-20 Micromol/L	5.8	6.0	-
CRP	0-5 mg/L	195	206.7	117
Procalcitonin	0-0.1 ng/mL	0.14	-	-
pH (Venous blood gas)	7.35-7.45	7.29 (L)	7.39	-
Lactate (Venous blood gas)	0.4-2.0 mmol/L	0.9	0.8	-
Total cholesterol	<5.2 mmol/L	Unreadable, see comment**	10.74	10.36
Triglycerides	<1.7 mmol/L	Unreadable, see comment**	15.75	11.18
HDL-C	>1.6 mmol/L	Unreadable, see comment**	0.48	0.49
LDL-C direct	<4.4 mmol/L	Unreadable, see comment**	2.80	3.13

**Figure 1:**
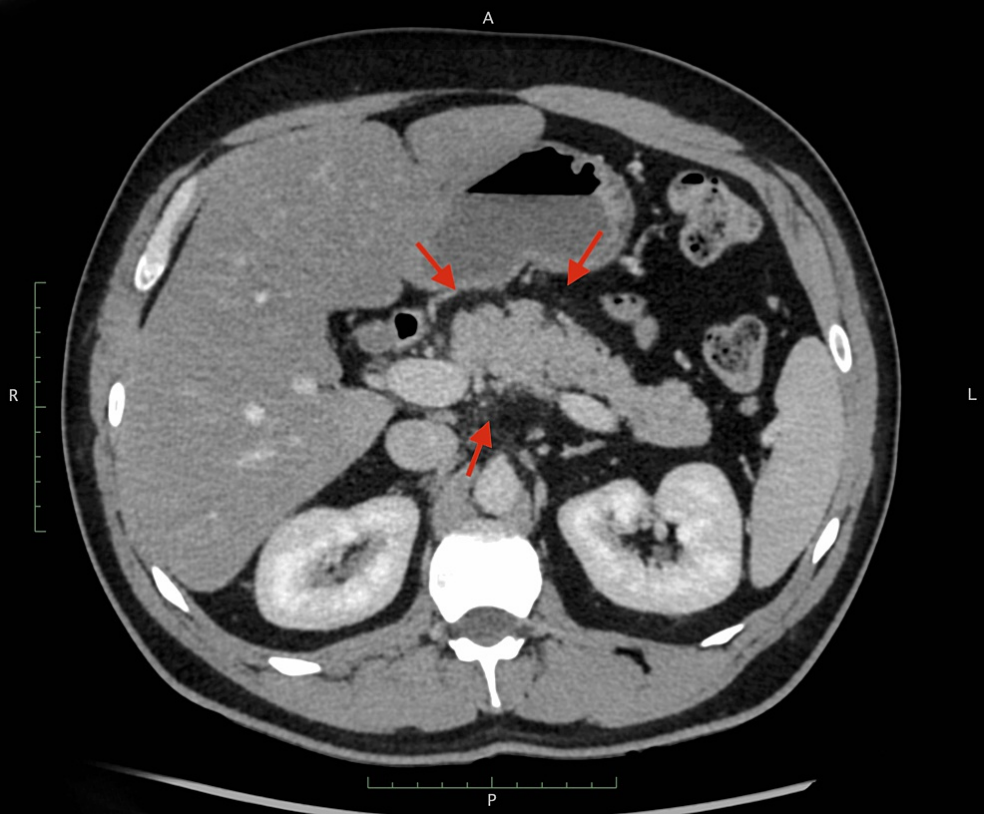
Contrast-enhanced CT scan of the abdomen showing subtle fat stranding around the pancreas (red arrows) consistent with pancreatitis CT: computed tomography

**Figure 2:**
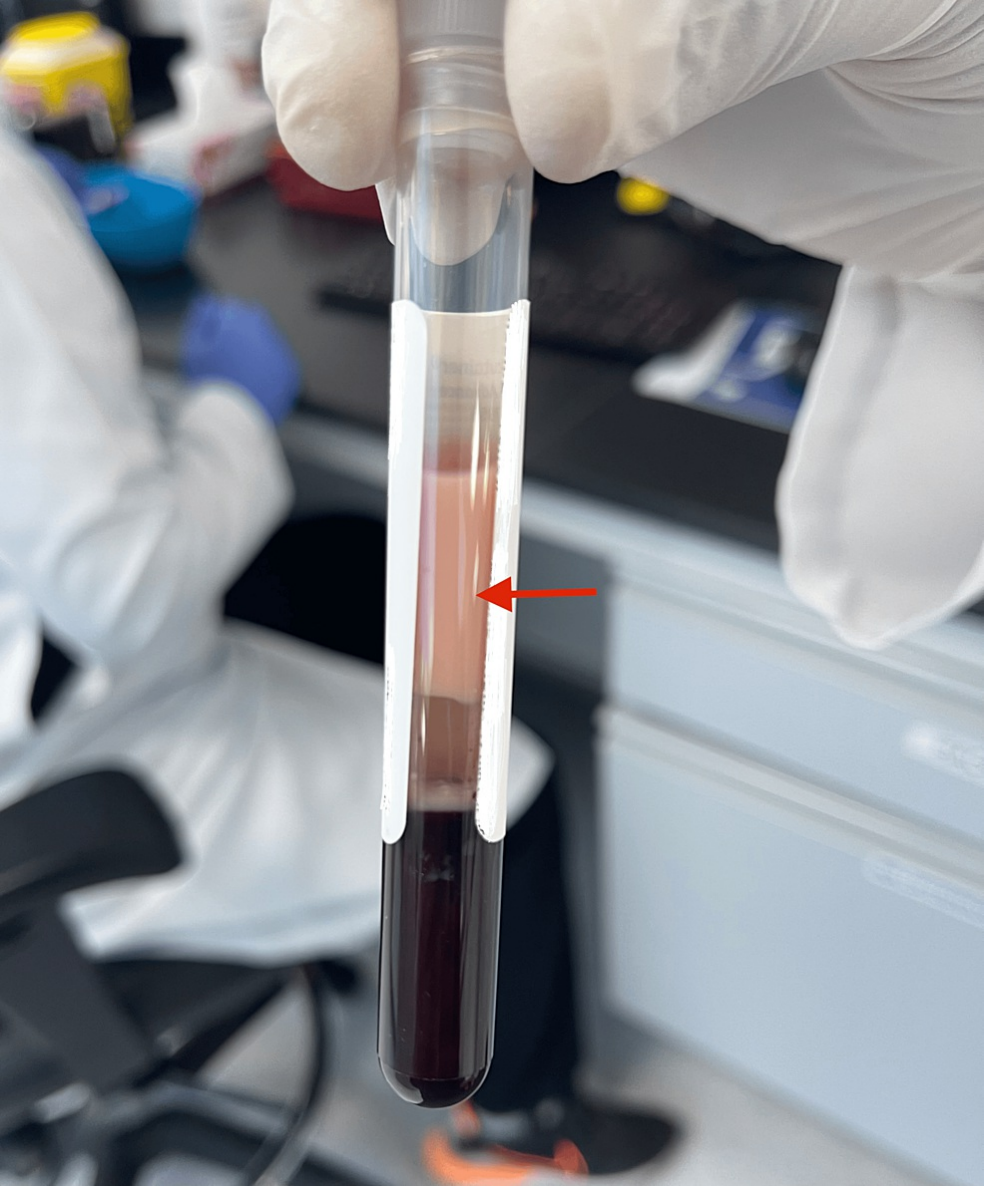
Patient's grossly "lipemic" serum (red arrow)

**Figure 3:**
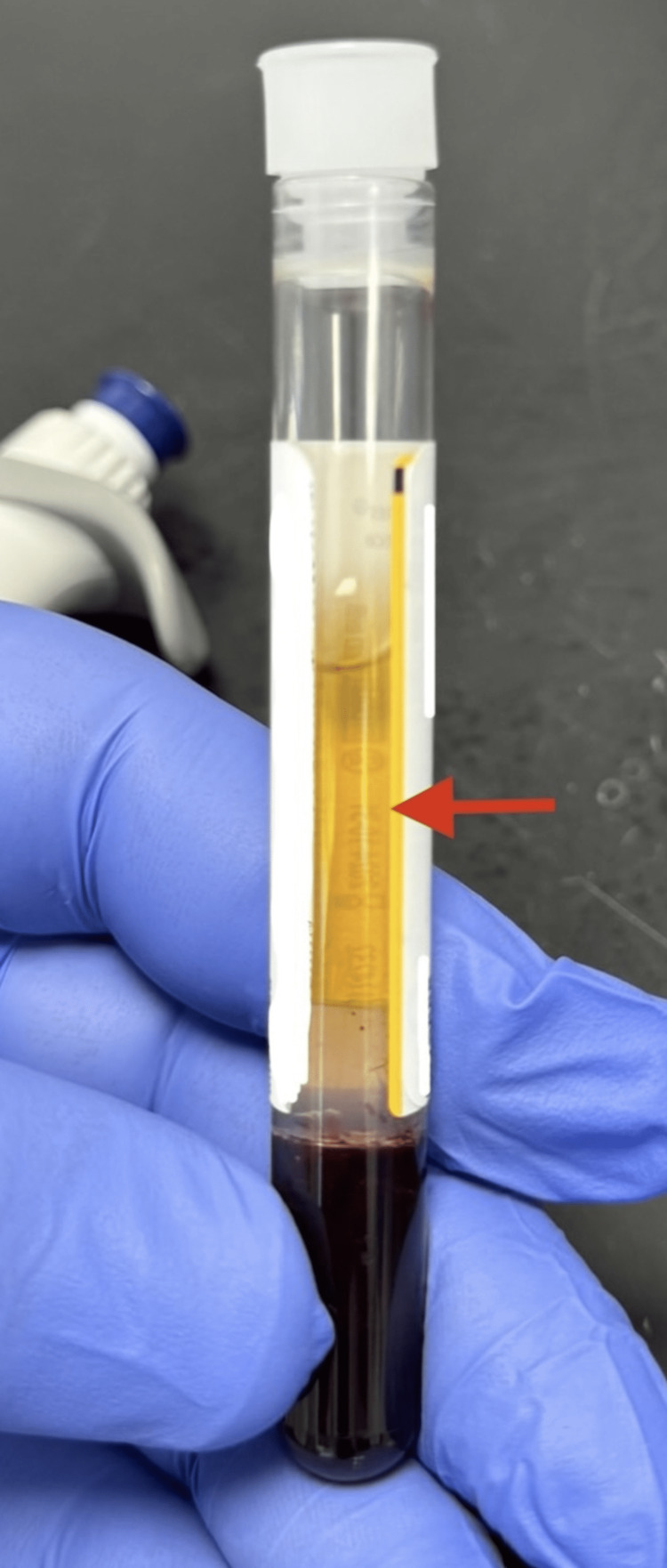
Normal serum (red arrow)

He was diagnosed as having euglycemic diabetic ketoacidosis (DKA), given the fact that he was on an SGLT2 inhibitor (dapagliflozin) known to be associated with the aforementioned condition; hence, the abdominal pain was attributed to it. He was managed accordingly, admitted, and started on a DKA protocol. He received IV fluids, insulin, and dextrose shortly after, after which his anion gap came down to 8 on subsequent venous blood gases. His abdominal pain was persistent despite the resolution of DKA; he was reassessed, and a CT abdomen was done, which showed evidence of fat standing around the pancreas (Figure 1), consistent with pancreatitis. His serum was reported to be grossly "lipemic" (Figure 2; see normal serum in Figure 3) on a routine blood test, and a lipid panel revealed markedly elevated triglyceride (TAG) levels (Table 1).

He was then diagnosed as having acute pancreatitis secondary to hypertriglyceridemia. He was kept on a fat-free diet and started on statins, and fibrates were introduced once serum TAG levels were below 11.3 mmol/l. All oral antiglycemics were kept on hold upon admission.

His serum TAGs were monitored, and they trended down to normal. His abdominal pain also resolved, and he was discharged without resuming sitagliptin and dapagliflozin pending review in the endocrinology clinic and proper hypertriglyceridemia control.

## Discussion

Acute pancreatitis is an important clinical condition. It is common, with an incidence ranging from 13 to 45 cases per 100,000 person-years [1]. It has an associated mortality rate of around 1-5% and approximately 20% of the cases recur [5]. It is most often accompanied by an elevation in pancreatic enzymes, with amylase and lipase being the most commonly tested and considered part of the diagnostic criteria [6]. The enzymes are elevated due to the destruction of the pancreatic parenchyma. The sensitivity and specificity of appropriately elevated amylase are 67-83% and 85-98%, respectively, and multiple studies have quoted the negative predictive value of a normal lipase to be between 94-100% [2,7]. Hence, suspicion is usually significantly reduced, or the diagnosis is even ruled out, when a patient's blood test comes back normal for these enzymes.

Although rare, acute pancreatitis can present with normal pancreatic enzymes, and according to the American College of Gastroenterology, the diagnosis of acute pancreatitis can be made when two out of the following three criteria are met: (1) characteristic abdominal pain, (2) serum amylase and/or lipase more than threefold the upper limit of normal, and (3) CT scan findings compatible with acute pancreatitis [8].

Acute pancreatitis with normal amylase and lipase levels can be attributed to several causes, some of which include: (1) Timing of the test. Serum amylase rises within 6 to 24 hours of the onset of acute pancreatitis. It has a short half-life of around 10 hours and, in uncomplicated attacks, returns to normal within three to five days. Serum lipase rises within four to eight hours of the onset of symptoms, peaks at 24 hours, and returns to normal within eight to 14 days. If blood tests are performed too early, they might yield normal results despite ongoing pancreatitis. Testing too late in the disease course can result in finding normal enzyme levels [9]. (2) Acute on chronic. Both amylase and lipase levels may not be elevated if there have been prior episodes of pancreatitis that have led to significant damage to pancreatic acinar tissue. It would preclude the release of expected amounts of enzymes during an acute attack due to the significant loss of pancreatic function and the replacement of pancreatic tissue by fibrous tissue [7,10]. (3) Alcohol. Elevations in serum amylase to more than three times the upper limit of normal may not be seen in approximately 20% of patients with alcoholic pancreatitis due to the inability of the parenchyma to produce amylase [11]. (4) Hypertriglyceridemia. Approximately 50% of patients with hypertriglyceridemia-associated pancreatitis have amylase and lipase levels under three times the upper limit of normal [12]. The serum of patients with hypertriglyceridemia may contain a circulating inhibitor that must be diluted before an elevation in serum amylase can be detected [7]. (5) Gallstone pancreatitis is another cause, but the mechanism is poorly understood.

In a paper by Omar et al. [13], a literature review of 26 patients with acute pancreatitis with normal lipase revealed the most common etiologies to be alcohol or unknown (6/26 patients each), gallstone pancreatitis (5/26 patients), and hypertriglyceridemia (4/26 patients) (Table 2).

**Table 2:** Summary of case reports of acute pancreatitis with normal lipase levels ALC: alcohol, HTG: hypertriglyceridemia, GST: gallstones, Unk: unknown/Unreported [13]

Patient characteristic	Details
Age range	26 to 84 years old
Day of presentation	6 (1 day), 14 (>1 day), 6 (Unk)
Gender	17 (Male), 8 (Female), 1 (Unk)
Diabetes mellitus	12
Etiology	6 (ALC), 5 (GST), 4 (HTG), 3 (Postop), 6 (Unk), 2 (others)
Death	2

In our case, the patient had hypertriglyceridemia and that was the cause of his normoenzymatic presentation. Hypertriglyceridemia is responsible for 1-7% of acute pancreatitis. A lipid panel should be checked as part of the workup, and a serum TAG level >5.6mmol/L is usually required before hypertriglyceridemia can be considered the underlying etiology [14]. He was also taking two medications known to be associated with pancreatitis: dipeptidyl peptidase-4 [15] and a sodium-glucose cotransporter-2 inhibitor [16], which could have worsened or triggered his symptoms given the background of undiagnosed hypertriglyceridemia. Although his hypertriglyceridemia was likely long-standing, the normal enzyme levels were less likely due to acute and chronic disease as he never had prior symptoms and there was no evidence of calcification on imaging.

## Conclusions

This case report underscores the importance of a comprehensive clinical assessment, including imaging, in cases where acute pancreatitis is suspected despite normal amylase and lipase levels. The traditional role of amylase and lipase as sensitive markers for pancreatitis may not apply to all cases, as we have discussed. A high index of suspicion, early recognition, and appropriate management are essential in preventing complications and improving patient outcomes in such scenarios. Physicians should also be cautious when prescribing medications associated with acute pancreatitis to patients with other risk factors such as uncontrolled diabetes, hypertriglyceridemia, etc.
